# Extemporaneous Cyclodextrin-Based Oral Solution of Ursodeoxycholic Acid Using a Ready-to-Use Vehicle

**DOI:** 10.3390/pharmaceutics18060734

**Published:** 2026-06-13

**Authors:** Antonio Lopalco, Oriana Boscolo, Annalisa Cutrignelli, Francesco Pio Cicinato, Sergio Fontana, Silvia Lucangioli, Nunzio Denora

**Affiliations:** 1 Department of Pharmacy—Pharmaceutical Sciences, University of Bari Aldo Moro, 4, E. Orabona Street, 70125 Bari, Italy; antonio.lopalco@uniba.it (A.L.); annalisa.cutrignelli@uniba.it (A.C.); f.cicinato@phd.uniba.it (F.P.C.); 2 Departamento de Tecnología Farmacéutica, Facultad de Farmacia y Bioquímica, Universidad de Buenos Aires, Junin 956, Buenos Aires C1113AAD, Argentina; oboscolo@ffyb.uba.ar; 3 Instituto de Tecnología Farmacéutica y Biofarmacia (InTecFyB), Facultad de Farmacia y Bioquímica, Universidad de Buenos Aires, Junin 956, Buenos Aires C1113AAD, Argentina; 4 Consejo Nacional de Investigaciones Científicas y Técnicas (CONICET), Godoy Cruz 2290, Buenos Aires C1414AAD, Argentina; 5 Centro Studi e Ricerche “Dr. S. Fontana 1900–1982”, Farmalabor s.r.l., 47, Piano S. Giovanni Street, 76012 Canosa di Puglia, Italy; s.fontana@farmalabor.it

**Keywords:** ursodeoxycholic acid, cyclodextrin, solubility, HPLC, pediatric, preformulation, formulation

## Abstract

**Background/Objectives:** Ursodeoxycholic acid (UDCA) is a bile acid widely used for the treatment of cholestatic liver diseases; however, its poor aqueous solubility represents a major limitation for the development of oral liquid formulations, particularly in pediatric patients requiring accurate and flexible dosing. This study aimed to develop and characterize a fully solubilized extemporaneous UDCA oral formulation using the ready-to-use vehicle Wagner, with particular emphasis on the role of hydroxypropyl-β-cyclodextrin (HP-β-CD) as a solubilizing excipient. **Methods:** Phase-solubility studies, Job’s plot analysis, and ^1^H NMR spectroscopy were performed to investigate the host–guest interaction between UDCA and HP-β-CD, confirming the formation of a stable 1:1 inclusion complex responsible for a marked increase in drug solubility. The aqueous solubility of UDCA increased from approximately 0.02 mg/mL in water to 31 ± 1 mg/mL in the Wagner base containing HP-β-CD, compared to ~10 mg/mL in the corresponding cyclodextrin-free vehicle. Chemical stability was evaluated using an HPLC method adapted from the European Pharmacopoeia, employing dual detection (refractive index and photodiode array detector) to ensure specificity and stability-indicating capability. **Results:** The UDCA solution (20 mg/mL) remained chemically stable for at least 4 months under refrigerated (4–8 °C) and room temperature (25 °C) conditions, with only moderate degradation observed at 40 °C. Physical stability studies confirmed the absence of precipitation, phase separation, or significant pH variations under all storage conditions. **Conclusions:** Wagner-based formulation enabled the development of a stable and homogeneous UDCA oral solution, providing a complementary formulation strategy to conventional suspension-based preparations. This approach represents a robust and patient-oriented strategy for extemporaneous compounding, particularly suitable for pediatric use.

## 1. Introduction

**Figure 1:**
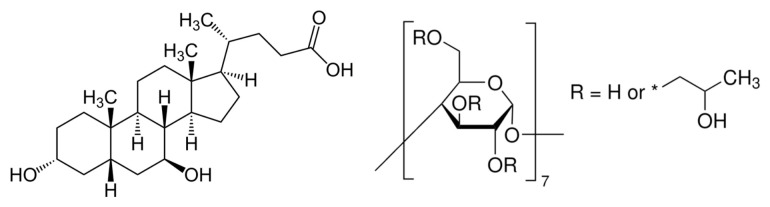
Molecular structure of ursodeoxycholic acid (UDCA) (**left**) and hydroxypropyl-β-cyclodextrin (HP-β-CD) (**right**). (*) denotes the substituent attached to the cyclodextrin.

Ursodeoxycholic acid (UDCA) (Figure 1) is a naturally occurring bile acid widely used in the treatment of cholestatic liver diseases, gallstone dissolution, and other hepatobiliary disorders [1,2,3].

Recent reviews and meta-analytical evidence have confirmed the continuing clinical interest in UDCA, including its effects on liver biochemical markers in metabolic dysfunction-associated steatotic liver disease and its use in gallstone prophylaxis after bariatric surgery [4,5].

Its therapeutic efficacy arises from its ability to promote bile flow and mitigate the cytotoxic effects of endogenous bile salts [1,3]. Despite its well-established clinical efficacy, UDCA is characterized by low aqueous solubility and a limited dissolution rate, which represent major constraints for its formulation, particularly in liquid dosage forms [6,7,8]. This poor biopharmaceutical profile often necessitates the use of enabling formulation strategies to achieve therapeutically relevant concentration in aqueous media.

The availability of liquid formulations is especially relevant in paediatric and geriatric populations, where dose flexibility, accurate administration, and patient acceptability are essential for therapeutic success [9,10,11].

Regulatory and professional bodies have repeatedly highlighted the lack of age-appropriate dosage forms as a critical unmet need, often requiring pharmacists to rely on extemporaneous compounding practices [12]. In the case of UDCA, commercially available liquid formulations are scarce, and hospital or community pharmacies frequently prepare oral suspensions using conventional dosage forms (capsules or tablets). However, such preparations may be affected by physical instability, sedimentation phenomena, poor dose uniformity, and limited palatability, potentially compromising both safety and adherence [9,11,13,14].

From a pharmaceutical technology perspective, the development of an oral solution of UDCA may represent a complementary formulation strategy to conventional suspensions, allowing homogeneous drug distribution, improved dose accuracy, and enhanced patient acceptability. Achieving this goal requires the use of liquid vehicles with adequate solubilizing capacity while maintaining acceptable viscosity, stability, and organoleptic properties. Ready-to-use liquid vehicles specifically designed for pharmaceutical compounding offer a practical and reproducible platform for this purpose, enabling standardized preparation procedures and reducing formulation variability in routine practice [15,16,17]. Among these, Wagner-based vehicle represents a promising approach due to their balanced composition, combining co-solvents, viscosity enhancers, buffering agents, solubilizing and taste-masking components. Specifically, the incorporation of hydroxypropyl-beta-cyclodextrin (HP-β-CD) could enable the delivery of poorly water-soluble drugs, thus enhancing their solubility. The Wagner ready-to-use vehicle was previously developed for pediatric oral compounding of a poorly water-soluble drug in the treatment of eosinophilic esophagitis. In particular, this vehicle was used for the preparation of pediatric budesonide oral solution, where the presence of HP-β-CD contributed to drug solubilization [16]. Based on these characteristics, this vehicle has been investigated as a suitable formulation platform for the preparation of UDCA oral solutions for pediatric patients.

Cyclodextrins (CDs) are well-established pharmaceutical excipients capable of improving the solubility, dissolution rate, and stability of poorly water-soluble drugs through the formation of inclusion complexes. Additionally, their ability to entrap molecules within their hydrophobic cavity can reduce direct interaction with taste receptors, thereby contributing to effective taste-masking of unpleasant or bitter drugs [18,19,20]. Among them, HP-β-CD is particularly attractive due to its high aqueous solubility, favourable toxicological profile, and proven effectiveness in oral liquid formulations [18,21,22]. Previous studies have shown that complexation with HP-β-CD can enhance the dissolution behaviour of UDCA, supporting its potential use in solution-based formulations [18,23]. However, a comprehensive correlation between complexation mechanism, solubilization efficiency, and formulation performance in ready-to-use vehicles remains insufficiently explored.

The phase-solubility method, introduced by Higuchi and Connors, provides a robust and established framework for characterizing drug/CD complexation and determining the apparent stability constant (K_1:1_) of the inclusion complex [24]. When combined with spectroscopic techniques such as ^1^H NMR, this approach enables not only quantitative assessment but also structural elucidation of host–guest interactions.

Within this context, the present study was designed using a sequential strategy guided by mechanistic considerations. Initially, the solubility behaviour of UDCA was investigated in aqueous environment in the presence of increasing concentrations of HP-β-CD in order to quantify the solubilising effects of the excipient, define the stoichiometric ratio of the drug/CD complex, and establish the basis for subsequent formulation development. Moreover, proton nuclear magnetic resonance (^1^H NMR) spectroscopy was employed to further elucidate the molecular interactions involved and to support hypotheses regarding the complexation mechanism.

Building on these findings, the role of CD was further investigated at the formulation level by comparing UDCA solubility in Wagner vehicle with and without HP-β-CD, thereby directly assessing its functional contribution within a pharmaceutically relevant system. This enabled the development of a fully solubilized oral formulation with drug concentrations compatible with flexible dosing while maintaining acceptable administration volumes for both pediatric and adult patients.

Finally, the chemical and physical stability of the optimized UDCA oral solution were evaluated under refrigerated (4–8 °C), room temperature (25 °C), and accelerated (40 °C) conditions. Chemical stability was assessed using an HPLC method adapted from the European Pharmacopoeia, employing dual detection (refractive index detector (RID), and photodiode array detector (DAD) to ensure specificity and reliable discrimination of UDCA from excipients and impurities. Physical stability was evaluated through visual inspection and pH monitoring.

This integrated analytical and formulation approach was intended to support the development of a stable, homogeneous, and patient-oriented UDCA oral solution, highlighting the critical role of CD complexation in enabling solubilization, stability, and palatability, and providing a mechanistically supported formulation approach complementary to traditional extemporaneous suspensions.

## 2. Materials and Methods

### 2.1. Materials

UDCA (Lot F2400123; Ph. Eur. grade; assay 100.8% on dried substance; molecular weight 392.6 g/mol) and fast oral solution “Wagner” (Lot W23009A) (composition: preserved water, HP-β-CD (molecular weight 1380, substitution degree equal to 1), carboxymethyl-cellulose sodium, sorbitol, glycerol, potassium sorbate, citric acid, trisodium citrate dihydrate, raspberry flavor) were obtained from FARMALABOR S.r.l (Canosa di Puglia, BT, Italy). Acetonitrile (Lot O2600; Honeywell, Seelze, Germany), Methanol (Lot P0550; Honeywell, Seelze, Germany), NaH_2_PO_4_ (Lot BCBJ8797V, Sigma-Aldrich, Milano, Italy), D_2_O and CD_3_OD (Sigma-Aldrich, Milano, Italy); Bidistilled water (Lot V5B605145B; Carlo Erba Reagents, Val de Reuil, France) were supplied by Levanchimica s.r.l. (Bari, Italy). Glass beakers, vials, graduate cylinders, pipettes, and volumetric flasks used were in borosilicate and were supplied by Levanchimica s.r.l.

### 2.2. Phase Solubility Studies and Determination of Inclusion Complex Constant

Phase solubility study was performed following the Higuchi–Connors method [24] to evaluate the aqueous solubility of the drug in the presence of increasing concentrations of HP-β-CD. Briefly, 2 mL samples containing aqueous solutions of HP-β-CD at six different concentrations, corresponding to 0, 1.25, 2.5, 5, 10, and 20% (*w*/*v*), (0, 8.34 × 10^−3^, 1.7 × 10^−2^, 3.3 × 10^−2^, 6.7 × 10^−2^, and 1.3 × 10^−1^ M, respectively), were prepared. An excess of UDCA was added to each solution to ensure saturation conditions.

The resulting suspensions were stored in an orbital shaker (MaxQ™ 6000 Incubated/Refrigerated Stackable Shakers; Thermo Fisher Scientific, Marietta, OH, USA) at 25 °C at a constant oscillation of 30 rpm for 72 h. After reaching equilibrium, the samples were centrifuged at 10,000 rpm for 15 min (Hettich MICRO22R centrifuge, Andreas Hettich GmbH & Co. KG, Tuttlingen, Germany), and the supernatant was analyzed after filtration through 0.22 µm cellulose acetate (CA) membrane filters (GVS North America, Sanford, ME, USA). The amount of solubilized drug in each sample was obtained by HPLC-UV.

Analyses using an Agilent 1260 Infinity II system (Agilent Technologies, Santa Clara, CA, USA) equipped with a Symmetry C18 column (5 µm, 4.6 mm × 150 mm; Waters Corporation, Milford, MA, USA) thermostated at 40 °C. The mobile phase consisted of acetonitrile and water (pH adjusted to 3 with phosphoric acid) in a 70:30 (*v*/*v*) ratio. An isocratic elution was employed at a flow rate of 1.0 mL/min, and the injection volume was 20 µL. Analyte detection was performed using a photodiode array detector (DAD) set at 205 nm. The expected retention time for UDCA was approximately 2 min [25]. Standard solutions were prepared from 20 mg/mL and 2 mg/mL methanolic stock solutions and further diluted to obtain concentrations ranging from 0.2 to 14 mg/mL. All solutions were filtered through 0.22 µm cellulose membrane filters before injection. Linearity was confirmed between 1.4 mg/mL and 14 mg/mL (R^2^ = 0.9977). The LOD and LOQ were 0.43 mg/mL and 1.29 mg/mL, respectively. Each sample was analyzed in triplicate.

Equation (1) was used to estimate the 1:1 inclusion constant of UDCA:HP-β-CD (K_1:1_):

K_1:1_ = p/S_0_(1 − p) (1)

where S_0_ is the intrinsic aqueous solubility of UDCA equal to 0.02 mg/mL as reported in the literature, and p is the slope of the phase solubility diagram. The phase-solubility diagram was constructed by plotting the equilibrium concentration of solubilized UDCA, expressed in molarity (M), against the corresponding molar concentration of HP-β-CD. Since the concentration of dissolved UDCA in the absence of HP-β-CD was below the limit of quantification (LOQ) of the analytical method, an S_0_ value of 0.02 mg/mL reported in the literature under the same experimental conditions [26] was used and converted into molarity. Linear regression analysis was performed using GraphPad Prism version 11.0.0 for macOS (GraphPad Software, Boston, MA, USA) on the mean values obtained from six points of the phase-solubility profile (*n* = 3). The regression was performed without constraining the intercept; therefore, the y-intercept represents the statistical intercept of the best-fit line and was not considered a measure of the intrinsic aqueous solubility of UDCA.

### 2.3. Job’s Plot

The stoichiometry of the inclusion complex between UDCA and HP-β-CD was investigated by the method of continuous variation (Job plot) [27].

Stock solutions of UDCA and HP-β-CD were prepared at the same molar concentration (2 × 10^−2^ M) in methanol/water (CH_3_OH:H_2_O, 7:3 *v*/*v*).

Different volumes of the two stock solutions were mixed to obtain eleven samples corresponding to UDCA molar fraction, r, ranging from 0.0 to 1.0 at intervals of 0.1, while maintaining the total molar concentration and the final volume constant for all samples. The UDCA molar fraction, r, was calculated according to Equation (2):

r = [UDCA]/([UDCA] + [HP-β-CD])(2)

where [UDCA] and [HP-β-CD] represent the molar concentrations of UDCA and HP-β-CD in each mixture, respectively. Therefore, r varied from 0 to 1, corresponding to solutions containing only HP-β-CD or only UDCA, respectively, with intermediate values representing different UDCA/HP-β-CD molar ratios. For each sample, the absorbance was measured at 205 nm. The absorbance variation associated with complex formation (ΔA) was calculated as the difference between the absorbance of the UDCA/HP-β-CD mixture and that of the UDCA alone in solution. The Job’s plot was then constructed by plotting ΔA × [UDCA] as a function of r. The position of the maximum of the curve was used to determine the stoichiometry of the inclusion complex.

### 2.4. Host–Guest Interaction Investigation by ^1^H NMR Study

To investigate the structural mechanism underlying the solubilization process and to verify the host–guest interaction at the molecular level between UDCA and HP-β-CD, ^1^H NMR spectroscopy was employed. Stock solutions of UDCA and HP-β-CD were prepared at the same molar concentration (5.01 × 10^−4^ M) in a mixture of deuterated methanol and deuterated water (CD_3_OD:D_2_O, 1:10 *v*/*v*). Subsequently, 350 μL of each stock solution were transferred into an NMR tube and mixed to obtain the UDCA/HP-β-CD system.

^1^H NMR spectra were acquired at 25 °C using an Agilent VNMRS 500 MHz spectrometer (Agilent Technologies, Santa Clara, CA, USA). Chemical shifts (δ) of the signals were expressed in parts per million (ppm), and all spectra were calibrated using the residual water signal (HDO) at 4.63 ppm as an internal reference to minimize experimental variability and allow reliable comparison among samples. The variation in chemical shift (Δδ) of the protons was calculated as the difference between the chemical shift in the hydrogens of UDCA in the presence of HP-β-CD and those of the UDCA alone.

### 2.5. Solubility of UDCA in the Vehicles

A preliminary solubility assessment of UDCA was carried out in the Wagner ready-to-use base and a Wagner-like vehicle with the same qualitative composition but devoid of HP-β-CD, supplied by Farmalabor upon request, to specifically elucidate the contribution of cyclodextrin to drug solubilization (Table 1). In the cyclodextrin-free vehicle, the amount of HP-β-CD (*w*/*w*) originally present in the Wagner base was quantitatively replaced with an equivalent weight of high purified water to maintain the same overall composition and mass balance of the vehicle.

An excess amount of UDCA (~40 mg/mL) was added to each vehicle and maintained under control stirring and temperature conditions (25 °C) until equilibrium was reached. The samples were then centrifuged to remove undissolved drug, and the supernatants were analyzed to determine the apparent solubility of UDCA. Solubility quantification was performed using an adapted HPLC method from the European Pharmacopoeia monograph for UDCA identification with minor modifications, to avoid the overlap of the peaks of the excipients present in the vehicles with the peak of UDCA [28].

HPLC analyses were performed using a Shimadzu Nexera system (Shimadzu Corporation, Kyoto, Japan) equipped with a refractive index detector (RID), a photodiode array detector (DAD) operating in 3D mode and a SIL-40C autosampler. Chromatographic separation was achieved on a Zorbax C18 column (250 × 4.6 mm, 5 µm; Agilent Technologies, Santa Clara, CA, USA) maintained at 40 °C. The mobile phase consisted of a mixture of 0.78 g/L NaH_2_PO_4_ buffer (pH adjusted to 3 with phosphoric acid), acetonitrile, and methanol in a 34:28:38 (*v*/*v*/*v*) ratio, delivered in isocratic mode at a flow rate of 1.0 mL/min. The injection volume was set at 50 µL. Under these conditions, UDCA eluted at approximately 13 min within a total runtime of 60 min. Detection was primarily performed using the RID, while the DAD supported peak purity assessment and analyte identification.

Calibration curve was constructed from a 3 mg/mL UDCA stock solution prepared in methanol and further diluted with the mobile phase to obtain working standard solutions. The method showed linearity in the concentration range of 3.0–0.40 mg/mL (R^2^ > 0.9999). The limit of detection (LOD) and the limit of quantification (LOQ) were determined to be 0.22 mg/mL and 0.66 mg/mL, respectively.

### 2.6. Physical-Chemical Stability Study

Following solubility assessment, UDCA formulation was prepared by using Wagner vehicle and subjected to chemical and physical stability studies. Six samples (volumes of 50 mL) were prepared at a concentration of 20 mg/mL of UDCA and stored in 60 mL amber glass containers away from light exposure at three different storage conditions as follows: 4–8 °C in a refrigerator, 25 °C in a thermostat chamber, and 40 °C and 75% RH in a climatic chamber (Climacell; MMM Medcenter Einrichtungen GmbH, Planegg, Germany). During a 4-month period, the chemical stability of UDCA in the samples was evaluated by the HPLC methods described in Section 2.5, quantifying the residual drug content and the eventual presence of degradants at several time points (0, 1, 3, and 4 months). The amount of drug and the possible presence of its degradants were assessed by analysing the chromatogram peaks and their areas in relation to the drug calibration curve. The chemical stability of UDCA was evaluated by periodically withdrawing aliquots of 1 mL from each sample, diluting them with methanol (1:10), and subjecting them to vortex mixing, sonication (2 min), and centrifugation (10 min at 25 °C, 13,200 rpm) to extract the drug from the precipitated polymer matrix. Aliquots of 1.0 mL of the supernatants were withdrawn and diluted 1:2 with the mobile phase prior to HPLC analysis. Drug content was determined with reference to the calibration curve. Physical stability of the formulations was assessed through visual inspection (turbidity, precipitation, colour changes, and phase separation) and pH monitoring during storage at 4 °C, 25 °C, and 40 °C for 4 months. The pH values were monitored using a METTLER TOLEDO SevenExcellence™ S400 Benchtop pH-meter (Mettler-Toledo GmbH, Greifensee, Switzerland).

## 3. Results and Discussion

The present study was designed to systematically investigate the feasibility of developing a fully solubilized oral formulation of UDCA using a ready-to-use vehicle, with particular emphasis on the role of HP-β-CD as a functional solubilizing excipient. To this end, an integrated analytical and formulation approach was adopted, combining phase-solubility analysis, spectroscopic characterization, and stability studies to elucidate both the mechanistic and technological aspects of the formulation. The experimental investigation was structured to first characterize the host–guest interaction between UDCA and HP-β-CD and its impact on aqueous solubility, and subsequently to translate these findings into a practical formulation context using the Wagner vehicle. Finally, the chemical and physical stability of the resulting oral solution was assessed under different storage conditions, with the aim of evaluating its suitability for extemporaneous compounding and potential application in clinical practice, particularly within the pediatric population.

### 3.1. Phase Solubility Studies and Determination of Inclusion Complex Constant

The phase-solubility profile of UDCA in the presence of increasing concentrations of HP-β-CD was determined after 72 h of equilibration at 25 °C. The experimental HP-β-CD concentrations used for the construction of the phase-solubility diagram and the corresponding equilibrium concentrations of solubilized UDCA are summarized in Table 2.

**Figure 2:**
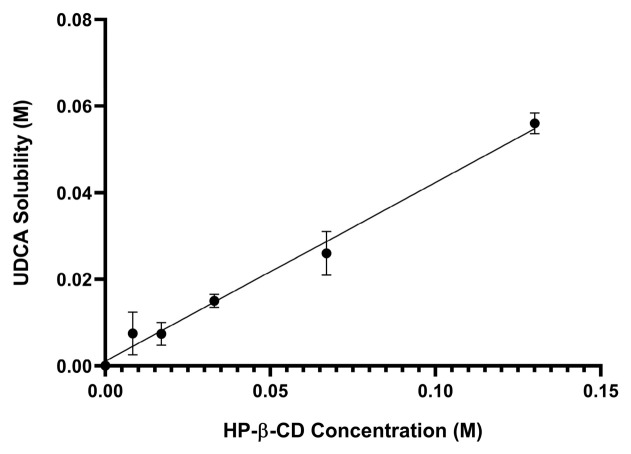
Phase–solubility diagram of UDCA with HP-β-CD at 25 °C after 72 h. Data are reported as mean ± SD (*n* = 3).

As illustrated in Figure 2, UDCA solubility increased as a function of HP-β-CD concentration, confirming the ability of the cyclodextrin to enhance the apparent aqueous solubility of the drug through complex formation.

The system exhibited a typical A_L_-type diagram according to the Higuchi–Connors model, characterized by a linear increase in drug solubility as a function of CD concentration. This behaviour is consistent with the formation of an aqueous soluble 1:1 inclusion complex and suggests the absence of significant higher-order complexation phenomena within the investigated concentration range.

The UDCA aqueous solubility increased markedly from the reported 0.02 mg/mL in water (5.1 × 10^−5^ M) up to ~22 mg/mL (0.056 M) in the presence of 20% (*w*/*v*) HP-β-CD (0.13 M) [26]. This represents an approximately 1100-fold increase in apparent solubility under the experimental conditions used in the phase-solubility study.

The relevance of this solubility enhancement is supported by recent formulation studies showing that UDCA still requires enabling strategies to overcome its poor dissolution and pH-dependent solubility behaviour [29,30].

The linear regression obtained after 72 h (Y = 0.4128X + 0.0011; R^2^ = 0.9905) showed excellent linearity, supporting a robust and reproducible complexation process. The apparent stability constant (K_1:1_), calculated from the slope according to the Higuchi–Connors method, was 13,784.9 M^−1^.

The apparent stability constant obtained for the UDCA/HP-β-CD complex was relatively high. This finding indicates a strong interaction between UDCA and HP-β-CD and explains the marked solubility enhancement observed in the phase-solubility study. However, highly stable drug/CD complexes may reduce the free drug fraction available for membrane permeation and potentially affect absorption, as reported for orally administered CD-based systems [31]. Nevertheless, CD inclusion complexes are dynamic and reversible systems, and drug release depends on the dissociation equilibrium as well as on dilution and absorption conditions [32]. Following oral administration, dilution in gastrointestinal fluids may shift the complexation equilibrium toward partial dissociation of the UDCA/HP-β-CD complex, promoting the release of free UDCA. Moreover, the solubility data of 10 mg/mL obtained with the Wagner-like vehicle (Table 3) suggests that UDCA solubilization in the final formulation is not exclusively dependent on HP-β-CD complexation, but also due to the contribution of other excipients [31].

### 3.2. Job’s Plot and Host–Guest Interaction Investigation by ^1^H NMR Study

**Figure 3:**
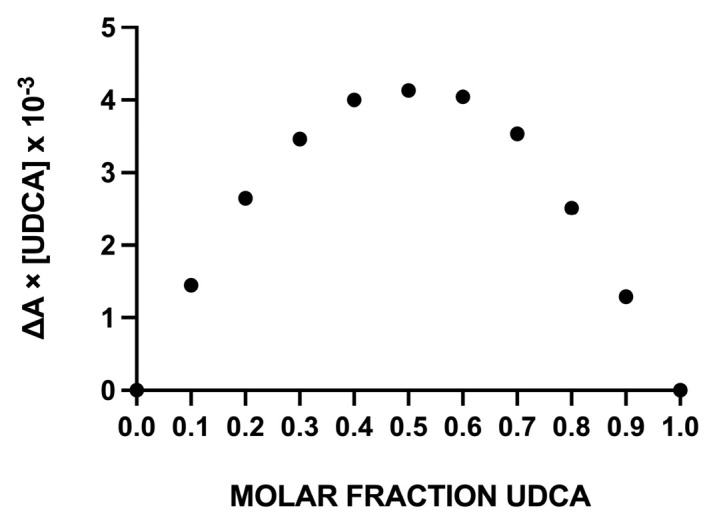
Job plot by UV-spectrophotometric analysis of the UDCA-HP-β-CD (5.01 × 10^−4^ M each in CH_3_OH/H_2_O, 7:3 *v*/*v*). The plot of ΔA × [UDCA] versus molar fraction of UDCA exhibits a maximum at X_(UDCA)_ ≈ 0.5, indicating a predominant 1:1 stoichiometry (UDCA:HP-β-CD).

The Job’s plot obtained by plotting ΔA × [UDCA] as a function of the UDCA molar fraction (Figure 3) showed a well-defined maximum at X_(UDCA)_ ≈ 0.5, clearly indicating a predominant 1:1 host–guest stoichiometry between UDCA and HP-β-CD in solution. This symmetric profile is characteristic of the formation of a single inclusion complex, in which one molecule of UDCA is accommodated within the hydrophobic cavity of one CD molecule. Unlike systems exhibiting deviations toward higher molar fractions, no significant shift in the maximum was observed, thus excluding the formation of higher-order complexes (2:1 or 1:2) under the investigated conditions. This behaviour is in full agreement with the phase-solubility analysis, which displayed an A_L_-type diagram consistent with 1:1 complex formation [27].

**Figure 4:**
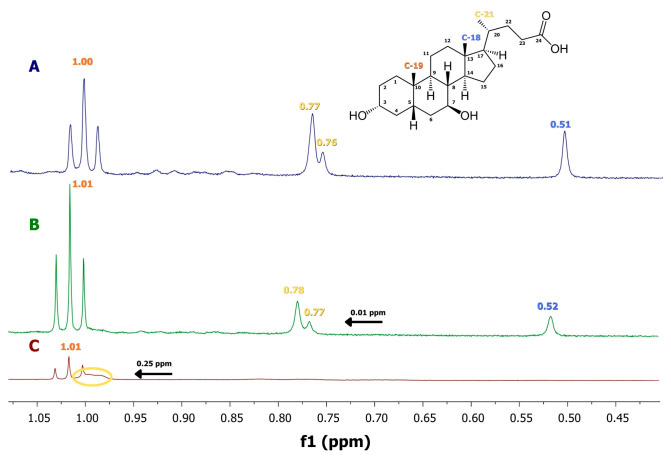
Superimposed ^1^H-NMR spectra (high-field region) highlighting the diagnostic methyl signals of UDCA. Top trace (blue, (**A**)): Free UDCA. Middle trace (green, (**B**)): UDCA and HP-β-CD at a 9:1 molar ratio. Bottom trace (red, (**C**)): UDCA and HP-β-CD at an equimolar (1:1) ratio.

This stoichiometric interpretation is further supported and structurally refined by the ^1^H NMR analysis, which provided direct molecular-level evidence of the inclusion process (Figure 4A–C). In the spectrum of free UDCA (Figure 4A), the characteristic methyl signals are clearly identifiable in the high-field region, with the H-18 angular methyl (ring D) appearing as a sharp singlet at approximately 0.51 ppm, the H-21 methyl group (side chain) resonating at approximately 0.76–0.77 ppm, and the H-19 angular methyl (ring A) falling at 0.99–1.01 ppm, where it overlaps with other signals [33]. It should be noted that HP-β-CD may also displays resonances in the high-field region, particularly the methyl protons of the hydroxypropyl substituents, which have been reported at approximately 1.1 ppm [34]. Therefore, the H-19 region of UDCA may be partially affected by overlapping with HP-β-CD signals.

Upon addition of HP-β-CD at a stoichiometric ratio 9:1, drug/CD, only slight downfield shifts were observed, indicating weak and transient interactions (Figure 4B). In contrast, at the equimolar ratio (1:1), corresponding to the maximum of the Job’s plot, a marked deshielding of the methyl signals was detected, confirming the formation of a stable inclusion complex within the cyclodextrin cavity (4 °C).

In particular, the H-18 signal exhibited the most pronounced complexation-induced shift, whereas the H-21 resonances showed smaller but significant variations. This differential behaviour provides key structural insight into the host–guest geometry and supports a “tail-in” inclusion mode, in which the hydrophobic side chain and ring D of UDCA penetrate deeply into the cyclodextrin cavity, while the more polar ring A remains partially exposed to the aqueous environment [6,35]. According to the model proposed by Mucci et al. [35], the greater shift observed for H-18 reflects its deeper positioning within the hydrophobic toroidal cavity, whereas H-19 remains closer to the wider rim of the cyclodextrin, resulting in a more moderate chemical shift variation.

Furthermore, the preservation of sharp and symmetrical signals, without peak broadening or splitting, indicates that UDCA remains chemically stable upon complexation and that the system is in fast exchange on the NMR timescale [18]. Overall, the excellent agreement between Job’s plot analysis, phase-solubility data, and NMR findings provides a coherent and robust interpretation of the system, confirming the formation of a stable 1:1 UDCA/HP-β-CD inclusion complex in solution. The identified inclusion geometry also offers a mechanistic explanation for the marked enhancement of UDCA solubility, as the partial inclusion of its hydrophobic steroidal moiety within the CD cavity effectively minimizes unfavorable interactions with the aqueous environment.

### 3.3. Solubility of UDCA in the Vehicles

The comparative solubility data clearly demonstrate the pivotal role of HP-β-CD in enhancing UDCA solubilization within the Wagner vehicle. In the absence of CD, the vehicle composed of glycerol and sodium carboxymethylcellulose was able to enhance UDCA solubility likely due to the combined effect of glycerin and the polymer up to approximately 10 mg/mL. However, this level remains insufficient for the preparation of highly concentrated and homogeneous liquid formulations. In contrast, the inclusion of HP-β-CD in the Wagner base enabled a marked increase in solubility, reaching approximately 30–32 mg/mL. This threefold enhancement confirms that CD is the key functional excipient, promoting the formation of a stable inclusion complex and allowing UDCA to remain fully dissolved.

To further clarify the contribution of the vehicle composition and pH to UDCA solubilization, the available solubility and pH data obtained in the investigated media are summarized in Table 3.

The Wagner and Wagner-like bases showed comparable slightly acidic pH values after UDCA addition and solubilization, namely 5.45 and 5.47, respectively. Therefore, the markedly higher solubility observed in the Wagner vehicle is consistent with the contribution of HP-β-CD to UDCA solubilization. By contrast, High purified water F.U. grade showed a higher pH value both before and after UDCA addition/solubilization, whereas the apparent solubility of UDCA remained extremely low. These findings indicate that pH alone was not sufficient to achieve therapeutically relevant UDCA concentrations under the tested conditions.

The relevance of the present formulation strategy should also be interpreted in light of recent studies on UDCA oral liquid preparations. Most published approaches have focused on suspension-based systems, confirming the clinical need for age-appropriate liquid formulations but also highlighting that conventional UDCA liquids are generally developed as dispersed systems. Boscolo et al. developed pediatric UDCA suspensions and evaluated their in vitro and in vivo performance, supporting the use of extemporaneous liquid preparations for pediatric administration [36]. Hausherr et al. described a high-strength UDCA oral suspension at 50 mg/mL intended for newborns [37]. More recently, Ip et al. reported the physicochemical and microbiological stability of compounded ursodiol oral suspensions prepared from different commercial sources in an oral suspending vehicle [38]. Together, these studies demonstrate the continued interest in UDCA liquid formulations, while also showing that suspension-based preparations require appropriate formulation, handling, redispersion, and quality control to ensure reliable administration.

From a clinical and technological perspective, the preparation of a solution becomes particularly relevant when compared with the extemporaneous suspension recommended by the Italian Society of Hospital Pharmacy (SIFO), typically prepared at a concentration of 60 mg/mL [39]. This higher nominal concentration may be useful when larger doses must be administered in limited volumes; however, as a heterogeneous dispersed system, the suspension requires adequate redispersion before administration and careful handling to minimize potential variability in dose uniformity, particularly in pediatric patients requiring accurate weight-based dosing [10,11,13,14].

In this context, the Wagner-based formulation developed in the present study should be considered as a complementary formulation strategy rather than as a direct replacement for existing suspension-based preparations. The main technological advantage of this approach is the achievement of complete drug solubilization within a ready to use vehicle, which may support homogeneous drug distribution and facilitate volumetric dose adjustment.

An additional relevant aspect concerns the excipient profile of the Wagner base and, in particular, the presence of HP-β-CD in a formulation intended for pediatric use. CDs are widely used functional excipients in drug delivery because they can improve the apparent aqueous solubility and chemical stability of poorly water-soluble drugs and may also contribute to taste masking [40,41]. In oral formulations, CDs generally show a favorable safety profile, mainly due to their limited gastrointestinal absorption and low systemic exposure [41,42].

Recent pediatric formulation studies have exploited HP-β-CD as an enabling excipient to develop oral liquid formulations with improved solubility, stability, and palatability. For example, Cirri et al. developed an HP-β-CD-based oral pediatric solution of propranolol, reporting improved photostability and taste masking, with a reduction in drug bitterness [43]. Similarly, the Wagner vehicle has previously been investigated as a ready-to-use oral compounding platform for pediatric formulations of poorly water-soluble drugs. In particular, Spennacchio et al. used this vehicle to prepare a pediatric mucoadhesive budesonide oral solution, where HP-β-CD contributed to drug solubilization and carboxymethylcellulose sodium provided viscosity-enhancing and mucoadhesive properties [16].

From a regulatory perspective, the EMA Q&A document on CDs used as excipients in medicinal products states that harmful effects of CDs are not expected at doses below 20 mg/kg/day [44].

Although the Wagner vehicle enabled UDCA solubilization up to approximately 30–32 mg/mL, a concentration of 20 mg/mL was kept below the experimentally observed solubility limit in order to provide a safety margin against possible precipitation during storage. Moreover, since UDCA therapy is generally based on body weight, a 20 mg/mL concentration was considered suitable to support flexible dose adjustment across different patient populations. In pediatric cholestatic patients, including neonates and infants, UDCA regimens in the range of 15–30 mg/kg/day have been reported, although cautious clinical use is recommended in this population [45]. In adults with primary biliary cholangitis, a dose of 13–15 mg/kg/day is recommended [46]. Neonatal formulary guidance also reports oral/intragastric use of UDCA for neonatal cholestasis, with weight-based dosing and a maximum daily dose of 30 mg/kg, further supporting the need for flexible oral liquid preparations in this population [47].

### 3.4. Physical-Chemical Stability Study

**Figure 5:**
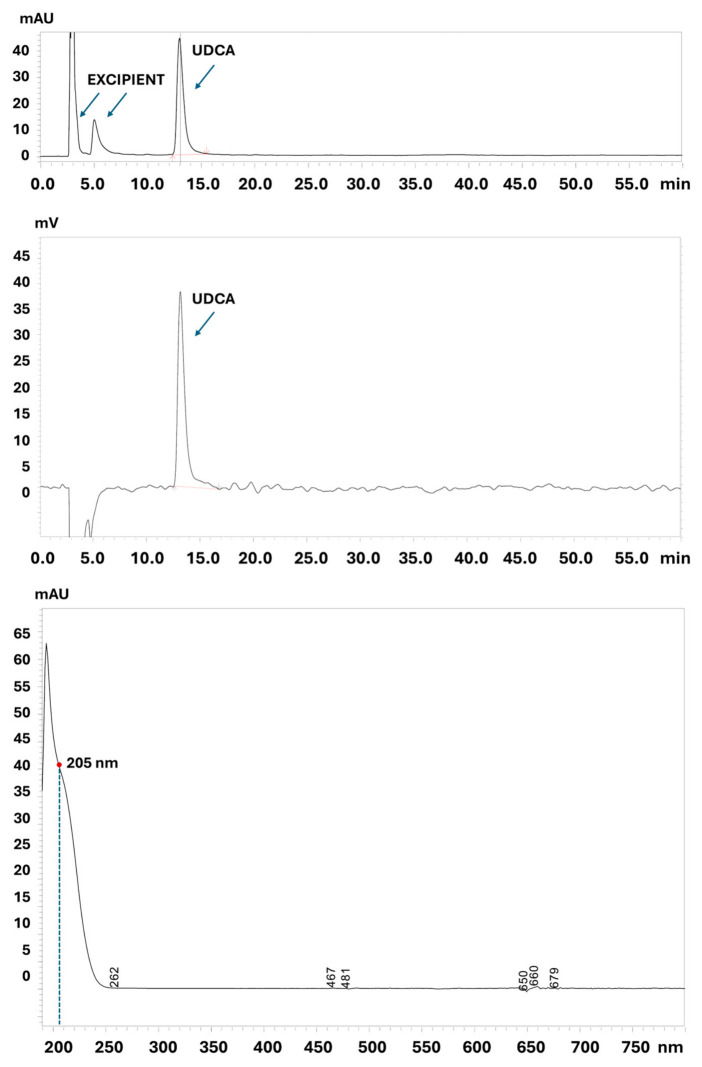
Representative HPLC chromatograms of UDCA sample recorded by DAD (**top**) and RID (**middle**) detectors. The identity of the drug was confirmed by its characteristic UV-Vis spectrum (**bottom**).

The evaluation of the chemical stability of UDCA in Wagner base required the implementation of a highly selective method capable of accurately quantifying the drug in the presence of a complex excipient matrix and impurities. To this end, an HPLC method adapted from the European Pharmacopoeia monograph was employed [28], integrating a dual-detection system based on a RID and a DAD (Figure 5).

Compared to conventional UV-based methods alone, the combined RID/DAD configuration provided improved selectivity and facilitated discrimination between the UDCA peak and formulation excipients. In addition, spectral analysis of the chromatographic peak by DAD confirmed its attribution to UDCA throughout the study. These features increased confidence in the quantification of UDCA and minimized the risk of analytical interference arising from the formulation matrix. The versatility of the method allowed its application across different stages of the study, including solubility determination in the vehicle and stability evaluation, thereby ensuring analytical consistency.

The chemical stability data of UDCA in the oral solution (Table 4) indicate a satisfactory stability profile over the 4-month study period, with a clear temperature-dependent behaviour. Under refrigerated conditions (4–8 °C), UDCA concentrations remained highly consistent, ranging from 19.36 to 20.52 mg/mL compared to the initial value of 20.62 mg/mL. The observed variations are minimal (within approximately ±6%) and do not follow any degradation trend, confirming excellent chemical stability and suggesting that low temperature effectively preserves the integrity of the drug in solution. At 25 °C, a moderate variability in UDCA content was observed. Concentrations decreased slightly over time, reaching 18.86 mg/mL at 3 months (approximately −8.5% relative to the initial concentration), before increasing again to 19.90 mg/mL at 4 months. Importantly, all values remained within the conventional ±10% acceptance range, indicating that the formulation maintains adequate chemical stability under room temperature conditions. The non-monotonic trend, particularly the increase at 4 months, suggests that the fluctuations are at least partially attributable to analytical variability rather than true degradation. Under accelerated conditions (40 °C), a more pronounced and progressive decrease in UDCA concentration was observed, with values declining to 18.52 mg/mL at 4 months (approximately −10.2% versus the initial concentration). This reduction approaches the typical acceptance limit, indicating the onset of potential thermally induced degradation processes. Nevertheless, the decrease remains relatively limited and gradual, suggesting that the formulation still provides a certain degree of protection even under thermal stress conditions. The absence of abrupt losses in drug content across all storage conditions supports the chemical robustness of the formulation. The presence of HP-β-CD likely could contribute significantly to this stability through inclusion complex formation, which can shield the hydrophobic core of UDCA from degradative pathways. Additionally, the buffered citrate system may help maintain a favourable pH environment, further limiting degradation. These results indicate that the UDCA in the formulation is chemically stable for at least 4 months under refrigerated and room temperature conditions, while exposure to elevated temperatures should be minimized to ensure optimal product quality.

**Figure 6:**
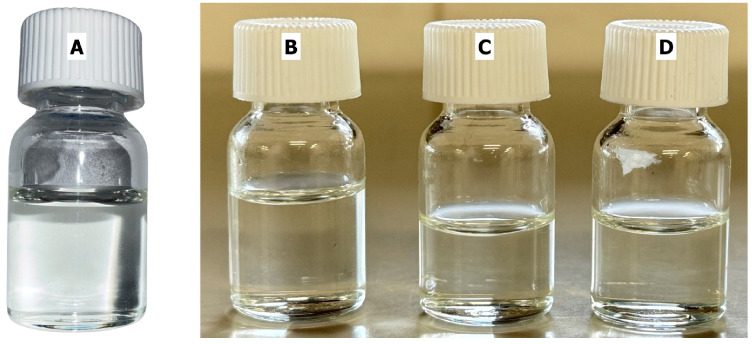
Visual appearance of the UDCA solution (20 mg/mL) formulated in fast oral solution Wagner vehicle at time zero (**A**) and after being stored in amber glass containers for 4 months at 25 °C (**B**), 40 °C (**C**) and 4 °C (**D**).

The physical stability of the UDCA oral solution formulated in Wagner vehicle was systematically evaluated during storage at 4, 25, and 40 °C, and the results demonstrated a highly robust and consistent profile. Throughout the entire study period, no visible signs of physical instability were observed in any of the samples. In particular, the formulations remained clear and homogeneous, with no evidence of turbidity, precipitation, colour changes, or phase separation when compared to their initial appearance (Figure 6).

This behaviour confirms that UDCA remained fully solubilized within the vehicle and that no recrystallization or phase transition phenomena occurred over time, even under accelerated conditions. In addition to visual inspection, pH monitoring further supported the physical stability of the system. The pH values remained essentially constant across all samples and storage conditions (variations < 0.5), indicating the absence of significant physicochemical changes within the formulation matrix. The stability of the pH is particularly relevant, as it reflects the buffering capacity of the citrate system and contributes to maintaining both drug solubility and chemical integrity.

These findings highlight the intrinsic advantage of the Wagner vehicle, in which the presence of HP-β-CD ensures thermodynamic stabilization of UDCA through inclusion complex formation, thereby preventing precipitation phenomena typically observed in supersaturated or poorly stabilized systems. This behaviour stands in contrast to conventional suspension formulations, where physical instability, such as sedimentation and aggregation, can compromise dose uniformity and overall product quality.

## 4. Conclusions

This study investigated the feasibility of developing a CD-based extemporaneous oral solution of UDCA using the Wagner ready-to-use vehicle. The proposed approach enabled the preparation of a fully solubilized UDCA formulation and may represent a complementary strategy to conventional extemporaneous suspensions, particularly in settings where homogeneous drug distribution and flexible dose adjustment are desirable. The work combines solubility enhancement strategies, physicochemical characterization, and stability assessment with a mechanistic understanding of CD-mediated complexation. From a formulation perspective, the results clearly highlight the important role of HP-β-CD as a key enabling excipient. The Wagner vehicle without CD showed a limited solubilizing capacity (~10 mg/mL), whereas the inclusion of HP-β-CD increased UDCA solubility up to approximately 31 mg/mL, representing a threefold enhancement. This effect was mechanistically supported by phase-solubility analysis, Job’s plot, and ^1^H NMR data, all consistently demonstrating the formation of a stable 1:1 inclusion complex responsible for the improved aqueous solubility and stabilization of the drug. The UDCA formulation (20 mg/mL) exhibited excellent chemical stability for at least 4 months under refrigerated (4–8 °C) and ambient (25 °C) conditions, with only moderate degradation observed at 40 °C. In parallel, the formulation showed outstanding physical stability, with no evidence of precipitation, turbidity, phase separation, or pH variation under any storage condition. This confirms the thermodynamic stability of the solubilized system and the protective role of cyclodextrin complexation within the buffered Wagner vehicle. The Wagner-based solution ensures homogeneous drug distribution, improved palatability (supported by the presence of sweeteners and flavouring agents), and accurate, flexible dosing, which are critical factors for neonates and young children. Moreover, the use of excipients commonly employed in oral formulations, combined with the functional role of HP-β-CD, supports the overall acceptability and applicability of the system in personalized therapy.

## Figures and Tables

**Table 1:** Qualitative Composition of Wagner ready-to-use base and Wagner-like vehicle.

Wagner Ready-to-Use Vehicle	Wagner-Like Vehicle
High purified water F.U. grade	High purified water F.U. grade
Hydroxypropyl-beta-cyclodextrin	-
Sorbitol	Sorbitol
Vegetable Glycerol E 422 PH. EUR.	Vegetable Glycerol E 422 PH. EUR.
Trisodium Citrate, Dihydrate	Trisodium Citrate, Dihydrate
Sodium carboxymethylcellulose	Sodium carboxymethylcellulose
Citric Acid	Citric Acid
Methylparaben	Methylparaben
Potassium Sorbate	Potassium Sorbate
Propylparaben	Propylparaben
Raspberry flavor	Raspberry flavor

**Table 2:** HP-β-CD concentrations used for the phase-solubility study and corresponding equilibrium concentrations of solubilized UDCA at the equilibrium at 25 °C. UDCA was added in excess to ensure saturation conditions.

HP-β-CD Concentration		UDCA Solubility	
(% *w*/*v*)	Molarity (M)	(mg/mL)	Molarity (M)
0	0	0.02 ^a^	5.1 × 10^−5^
1.25	8.34 × 10^−3^	2.94 ± 1.92	7.5 ± 4.9 × 10^−3^
2.5	1.7 × 10^−2^	2.90 ± 1.02	7.4 ± 2.6 × 10^−3^
5	3.3 × 10^−2^	5.89 ± 0.59	1.5 ± 0.15 × 10^−2^
10	6.7 × 10^−2^	10.21 ± 2.00	2.6 ± 0.51 × 10^−2^
20	1.30 × 10^−1^	21.98 ± 0.94	5.6 ± 0.24 × 10^−2^

^a^ value from the literature [26].

**Table 3:** Summary of UDCA solubility and pH values in water and the investigated media. pH values were measured before and after UDCA addition; UDCA was added at a nominal concentration of 20 mg/mL for pH evaluation.

Vehicle	UDCA Solubility (mg/mL)	pH Without UDCA	pH with UDCA ^b^
High purified water F.U. grade	0.02 ^a^	7.05	5.96
Wagner base	31.0 ± 1.0	5.77	5.45
Wagner-like base	~10	5.58	5.47

^a^ data from the literature [26]; ^b^ pH values determined at the UDCA equilibrium solubility after adding 20 mg/mL of UDCA.

**Table 4:** Chemical stability of UDCA oral formulations (20 mg/mL) prepared in Wagner ready-to-use vehicle stored at 4–8, 25 and 40 °C, determined by HPLC-RID over 4 months. Results are expressed as mean concentration ± S.D. (mg/mL), *n* = 3 and recovery (%) with respect to the UDCA content at time 0 are summarized.

Temperature (°C)	Time (Month)	UDCA Concentration ± S.D. (mg/mL)	Recovery (%)
4–8	0	20.62 ± 0.32	100.0 ± 1.55
	1	19.49 ± 0.59	94.5 ± 2.86
	2	19.65 ± 0.46	95.3 ± 2.23
	3	20.52 ± 0.28	99.5 ± 1.36
	4	19.36 ± 0.70	93.9 ± 3.39
25	0	20.62 ± 0.32	100.0 ± 1.55
	1	19.20 ± 0.90	93.1 ± 4.36
	2	19.28 ± 1.07	93.5 ± 5.19
	3	18.86 ± 0.96	91.5 ± 4.66
	4	19.90 ± 0.24	96.5 ± 1.16
40	0	20.62 ± 0.32	100.0 ± 1.55
	1	19.38 ± 1.01	94.0 ± 4.90
	2	19.33 ± 1.04	93.7 ± 5.04
	3	18.66 ± 0.96	90.5 ± 4.66
	4	18.52 ± 0.24	89.8 ± 1.16

## Data Availability

The original contributions presented in this study are included in the article. Further inquiries can be directed to the corresponding author.
